# Latent virus reactivation in astronauts on the international space station

**DOI:** 10.1038/s41526-017-0015-y

**Published:** 2017-04-12

**Authors:** Satish K. Mehta, Mark L. Laudenslager, Raymond P. Stowe, Brian E. Crucian, Alan H. Feiveson, Clarence F. Sams, Duane L. Pierson

**Affiliations:** 1 Jestech, Johnson Space Center, NASA, Houston, TX 77058 USA; 2 University of Colorado Denver, Anschutz Medical Campus, 12700 E. 19th Ave, Aurora, CO 80045 USA; 3 Microgen Laboratories, 903 Texas Ave, La Marque, TX 77568 USA; 4 NASA Johnson Space Center, Mail code SK, 2101 NASA Parkway, Houston, TX 77058 USA

## Abstract

Reactivation of latent herpes viruses was measured in 23 astronauts (18 male and 5 female) before, during, and after long-duration (up to 180 days) spaceflight onboard the international space station . Twenty age-matched and sex-matched healthy ground-based subjects were included as a control group. Blood, urine, and saliva samples were collected before, during, and after spaceflight. Saliva was analyzed for Epstein–Barr virus, varicella-zoster virus, and herpes simplex virus type 1. Urine was analyzed for cytomegalovirus. One astronaut did not shed any targeted virus in samples collected during the three mission phases. Shedding of Epstein–Barr virus, varicella-zoster virus, and cytomegalovirus was detected in 8 of the 23 astronauts. These viruses reactivated independently of each other. Reactivation of Epstein–Barr virus, varicella-zoster virus, and cytomegalovirus increased in frequency, duration, and amplitude (viral copy numbers) when compared to short duration (10 to 16 days) space shuttle missions. No evidence of reactivation of herpes simplex virus type 1, herpes simplex virus type 2, or human herpes virus 6 was found. The mean diurnal trajectory of salivary cortisol changed significantly during flight as compared to before flight (*P* = 0.010). There was no statistically significant difference in levels of plasma cortisol or dehydoepiandosterone concentrations among time points before, during, and after flight for these international space station crew members, although observed cortisol levels were lower at the mid and late-flight time points. The data confirm that astronauts undertaking long-duration spaceflight experience both increased latent viral reactivation and changes in diurnal trajectory of salivary cortisol concentrations.

## Virus reactivation in long-duration spaceflight

Long-duration spaceflight increases the reactivation of latent herpes viruses in astronauts and is accompanied by a rise in stress hormone levels. This study shows that the frequency and viral loads of reactivation of Epstein-Barr virus, varicella-zoster virus, and cytomegalovirus were even greater in blood, urine, and saliva samples from astronauts staying 60 to 180 days onboard the International Space Station than has previously been observed for short-duration (10–16 days) missions. Changes in viral reactivation were also found to be associated with changes in the daily trajectory of salivary cortisol during these long-duration missions. These results indicate that the effects of the microgravity environment on the immune system are increased with prolonged exposure and highlight the potential increased risk of infection among crewmembers.

## Introduction

Increased reactivation of some naturally occurring latent herpes viruses including Epstein–Barr virus (EBV), varicella-zoster virus (VZV) and cytomegalovirus (CMV) was previously demonstrated in astronauts during short-duration (10–16 days) space shuttle flights.^1^ However, following reactivation, viruses were shed in body fluids and the astronauts were typically asymptomatic.^2^ Stress responses associated with spaceflight include activation of the hypothalamic-pituitary-adrenal and the sympathetic-adrenal-medullary axes ^3^ and may result in the reactivation of latent herpes viruses subjecting astronauts to risk of shedding live, infectious viruses during spaceflight.^4, 5^ Cortisol and dehydoepiandosterone (DHEA) may affect regulation of cellular immunity resulting in reactivation of latent viruses.^4^ Responses to reactivation of herpes viruses can be asymptomatic, debilitating, or even life-threatening. Isolation of crewmembers before flight has no mitigating effect on latent virus reactivation. Even a complete quarantine does not prevent viral reactivation during spaceflight.^5^ The goal of this study was to determine whether long-duration (up to180 days) spaceflight aboard the international space station (ISS) would allow astronauts to acclimate to spaceflight and mitigate the impact of spaceflight associated stressors on crewmembers.^6^ In the present study, viral reactivation and shedding of EBV, VZV, CMV, HSV1, and human herpes virus 6 (HHV6) were measured in 23 US astronauts before, during, and immediately following long duration spaceflight. Recently, immune changes were shown to persist during and after long-duration ISS missions in the same participants as studied in the present virus study.^7^ In an environment of immune dysfunction, our hypothesis is that viral reactivation and shedding of these herpes viruses will also increase in astronauts during long-duration space flight as observed in ground-based space analogs.^8^

## Results

### Viral reactivation

**Fig. 1:**
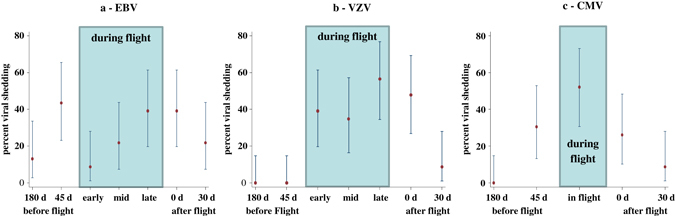
Binomial 95% confidence intervals for percentage of crewmembers shedding virus, **a**: EBV, **b**: VZV, **c**: CMV

Twenty-two of 23 astronauts shed one or more target viruses (Table 1). Fifteen astronauts shed VZV, 22 shed EBV, and 14 shed CMV at one or more time points before, during, or after spaceflight (Table 1). One astronaut did not shed any virus during any defined collection time. By contrast, none of the 20 control subjects shed VZV or CMV and only 2 of them shed EBV (Table 1). No astronauts or control subjects shed HSV1, HSV2, or HHV6 at any time throughout the study. Percent shedding among crewmembers with 95% binomial confidence intervals are shown for EBV, VZV, and CMV in Fig. 1. For these three viruses, there was considerable variation of the shedding percentages over the collection time points (Fig. 1) suggesting a possible overall mission effect on the reactivation of these viruses. More formally, when comparing copy numbers between time points, the Friedman test did not show a significant difference between time points for EBV (*P* = 0.064). Indeed, EBV was shed at all seven sample collection time points (Two before, three during, and two after flight). For VZV, no shedding occurred at both 180 days and 45 days before flight but shedding was found in early, mid, and late time points during flight as well as at landing and 30 days after landing (Table 1). The Friedman test comparing copy number distributions was significant for VZV (*P* < 0.0001). After adjustment for multiple comparisons, we found significantly more VZV reactivation in the late-flight time period than either pre-flight (*P* = 0.011) or 30d-post-flight (*P* = 0.035). There was no CMV shedding at 180 days before flight but there was CMV shedding 45 days before, during, and after flight. The Friedman test comparing copy numbers for CMV was also significant (*P* < 0.0001). In particular, also after adjustment for multiple comparisons, we found that this was due to increased CMV shedding during flight which was significantly greater than 180 days before flight (*P* = 0.013) and also greater than 30 days after flight (*P* = 0.041). Even when the time points with no shedding were excluded from the analysis, there were still significant differences between the remaining time points; *P* = 0.0027 (VZV) and *P* = 0.0008 (CMV) (Fig. 1).

**Table 1:** Salivary VZV, Salivary EBV and urinary CMV copies in the 23 international space station crewmembers before, during and after the flight

S. No.	VZV copies/ng salivary DNA							EBV copies/ng salivary DNA							*CMV copies/ng urinary DNA				
	Before Flight		During Flight			After Flight		Before Flight		During Flight			After Flight		Before Flight		During Flight	After Flight	
	L-180	L-45	Early	Mid	Late	R+0	R+30	L-180	L-45	Early	Mid	Late	R+0	R+30	L-180	L-45		R+0	R+30
1	0	0	368	0		0	0	0	0	0	640		128	0	0	0	0	0	0
2	0	0	45	0	816	660	606	0	0	0	0	630	88	0	0	0	450	0	0
3	0	0	0	0		0	0	0	0	0	0		98	0	0	0	300	0	0
4	0	0	816	0	482	220	0	0	0	0	450	770	65	0	0	0	250	40	0
5	0	0	60	0	1300	0	0	87	0	0	0	321	70	0	0	48	50	90	0
6	0	0	61	0	480	180	0	0	150	0	0	0	71	0	0	136	0	120	89
7	0	0	0	0	0	0	0	0	34	0	0	0	102	0	0	45	120	70	0
8	0	0	130	380	560	0	0	0	110	100	0		0	126	0	345	0	0	0
9	0	0	0	0		0	0	89	0	0	1020	1215	0	120	0	0	400	0	0
10	0	0	0	0	0	0	0	0	46	0	0	687	0	117	0	0	0	0	0
11	0	0	0	370	570	0	0	0	143	0	0	814	0	390	0	0	350	0	0
12	0	0	200	290	110	0	0	0	98	0	0	900	0	0	0	0	0	0	0
13	0	0	0	0		60	0	66	0	0	0	0	0	0	0	0	0	0	0
14	0	0		0	69	125	0	0	68		0	400	496	0	0	0	560	0	0
15	0	0		0	0	0	0	0	0		0	0	0	0	0	0	0	0	0
16	0	0		0	120	300	0	0	0		498	0	0	0	0	0	0	0	0
17	0	0	160	180	150	230	0	0	81	0	0	0	0	0	0	57	378	0	0
18	0	0	0	156	0	155	0	0	0	0	0	400	0	0	0	0	0	0	0
19	0	0	0	276	150	0	0	0	0	0	0	0	350	0	0	0	0	0	0
20	0	0	280	340	356	200	0	0	0	0	490	0	0	0	0	56	467	0	340
21	0	0	0		414	120	63	0	55	0		0	0	0	0	0	0	0	0
22	0	0		0	0	0	0	0	50		0	0	0	0	0	60	80	40	0
23	0	0		0	0	0	0	0	0		0	0	0	487	0	0	790	450	0

*L* Launch of spaceflight, *R* return of spaceflight

*Notes:* Highest copy number of the four samples taken at each time point was given in this Table

There was only one urine sample inflight, hence only one CMV measurement is shown. Blank cells indicate no sample was collected

**Table 2:** Number of crewmembers shedding viruses in their saliva before, during and after International Space Station and Space Shuttle missions

	VZV						EBV						CMV					
	Any TimeDuring Mission	B	During Flight			A	Any TimeDuring Mission	B	During Flight			A	Any TimeDuring Mission	B	During Flight			A
			E	M	L				E	M	L				E	M	L	
ISS *N = 23	15 (65%)	0	9	7	13	10	22 (96%)	13	1	5	9	14	14 (61%)	7	4	5	3	6
SS **N = 17	7 (41%)	0	7	NA	NA	4	14 (82%)	10	14	NA	NA	10	8 (47%)	4	6	NA	NA	4

*B* Before, *A* After, *E* Early, *M* Mid, *L* Late during the flight, *ISS* International Space Station, *SS* Space Shuttle

*N = 23 current study

**N = 17 previous study, see ref. 1

**Fig. 2:**
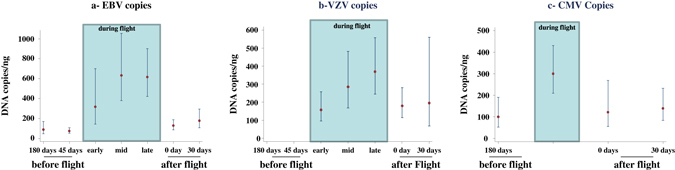
Estimated median viral copies given that shedding has occurred, with 95% confidence intervals (mixed-model regression). **a**: EBV, **b**: VZV, **c**: CMV

Viral copy numbers for positive tests had skewed distributions, so we show results in terms of medians rather than means. For the same viruses as above, Figure 2 shows median number of copies for EBV, VZV, and CMV along with 95% confidence limits obtained from mixed-model regression analysis. The overall mission effect on copy numbers was evident for EBV *(P* < 0.001), VZV (*P* = 0.057), and CMV (*P* = 0.001). For EBV, post-hoc comparisons with Sidak-adjusted *P*-values reflected higher median viral copies during the last two flight periods (Mid, Late) relative to pre-flight (*P* < .001, both comparisons) and relative to 30 days post-flight (*P* < 0.001, both comparisons). Median copies for early recovery (at landing) were not significantly higher than preflight (unadjusted *P* = 0.08) (Fig. 2). For CMV, the median copy number was significantly higher during flight than before flight (*P* < 0.001), but not for landing day (unadjusted *P* = 0.56) or 30 days after landing (unadjusted *P* = 0.55). No post-hoc comparisons were made for median copies of VZV because the overall mission effect was not significant.

### EBV DNA levels in peripheral blood mononuclear cells (PBMCs)

**Fig. 3:**
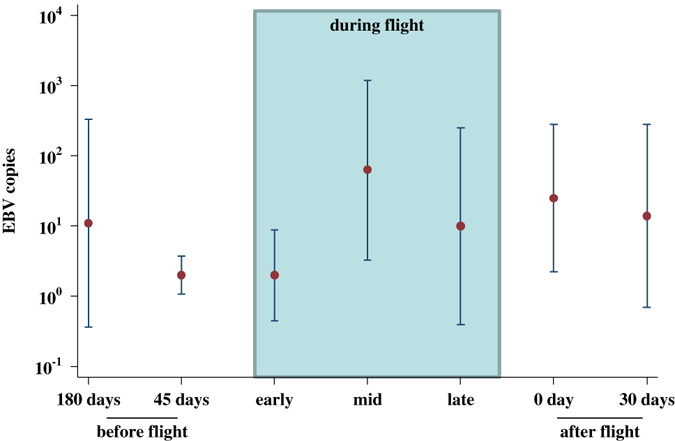
Median Levels of EBV DNA (with 95% confidence limits) in peripheral blood mononuclear cells (PBMCs) before, during, and after long-duration spaceflight

EBV DNA levels varied considerably: from below or at the detectable limit of 2 copies (55 cases) to a maximum of 71,500 copies, with 25 instances of at least 1000 copies being detected. Because of this high degree of variation, we did not find a significant difference between median copy levels over the time points as a whole (*P* = 0.21, median regression analysis), although it did appear that median copies were somewhat elevated at the later time points (Fig. 3).

### Plasma cortisol

**Fig. 4:**
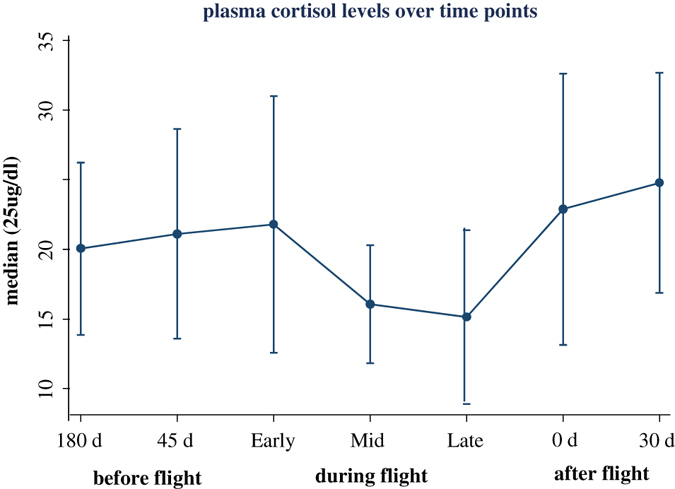
Estimated medians and 95% confidence limits for plasma cortisol

Median plasma cortisol levels ranged from 15.2 μg/dL (late flight) to 24.8 μg/dL (late recovery). The overall test for differences in medians between the seven time points was not significant (bootstrapped median regression: *P* = 0.086). Estimates of medians and 95% confidence limits for each time point are shown in Fig. 4.

### Salivary cortisol and DHEA

**Fig. 5:**
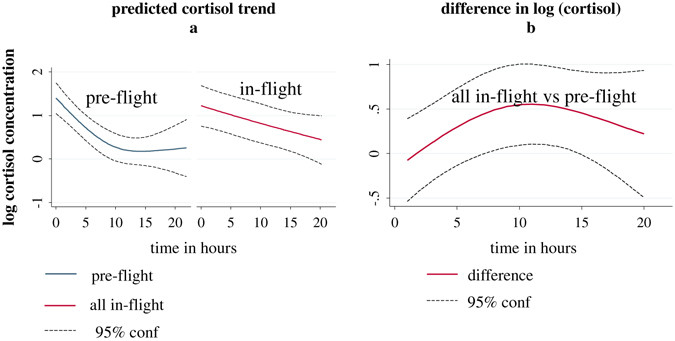
Predicted mean log cortisol concentration trend and 95% confidence intervals for before-flight and during-flight phases in (**a**). **b** shows the difference between the two phases as well as the 95% confidence interval. It can be seen that the greatest difference occurs at about 10 h after waking based on these estimations

**Fig. 6:**
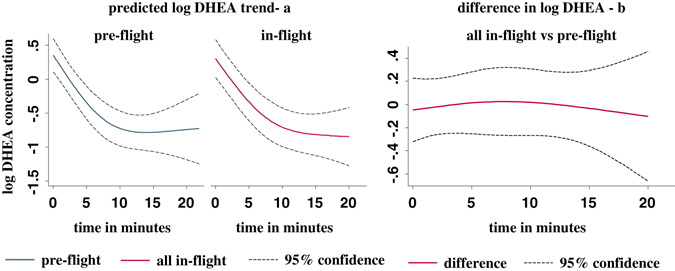
Predicted mean log DHEA trend and 95% confidence intervals for before-flight and during-flight phases in (**a**). **b** shows the difference between the two phases as well as the 95% confidence interval

With natural log cortisol concentration as outcome, there was some evidence that daily mean trajectories for each of the three during-flight periods differed from the trajectories before flight (unadjusted *P* = 0.069, 0.022, 0.086 by mixed-model regression on log cortisol concentration values for early-flight vs. before-flight, mid-flight vs. before-flight, and late-flight vs. before-flight, respectively). However, the flight periods did not differ significantly between themselves (*P* = 0.132). After combining data across the three flight periods and comparing the mean daily trajectory during flight with pre-flight, we found evidence for an effect of flight (*P* = 0.010, see Appendix). With one outlier subject removed (see details in the Appendix), there was even stronger evidence of a flight effect (*P* = 0.0015). Figure 5a shows the predicted mean daily trajectory of log cortisol concentration before flight and during flight with 95% confidence limits. The difference was greatest around the middle of the waking period (about 10 h). A plot of this difference vs. hours since awakening with 95% confidence limits is shown in Fig. 5b. There was no evidence that the mean cortisol trajectory during the first period after flight was different from pre-flight (*P* = 0.42). There was some evidence of a difference between the second recovery period and before flight (unadjusted *P* = 0.048) (see Discussion). Estimated diurnal declines of DHEA did not change as a function of study phase. Figure 6a shows these trends (on a log scale) for the before and during flight periods and Fig. 6b shows the estimated difference in these trends between the “during flight” and “before flight” periods.

### Anti-viral antibodies

After adjusting for differences between subjects, titer levels of anti-EBV viral capsid antigen (VCA) antibodies appeared increased relative to the first pre-flight period. However, because of high variability we found no significant difference between the periods (*P* = 0.53, repeated measures ordered logit analysis). Similarly we could not detect any effect of flight on anti-CMV VCA antibodies (*P* = 0.79).

## Discussion

This is the first study of reactivation of multiple latent herpes viruses in astronauts during long-duration spaceflight (60–180 days). We have previously shown a direct correlation of immune changes and viral reactivation in a study of 17 astronauts during short-duration (10–16 days) spaceflight.^1^ In those subjects, substantial changes in cell-mediated immunity were found in most astronauts that reactivated one or more of the target herpes viruses.^1, 7^ Glaser^9^ previously showed association of EBV reactivation and diminished cell-mediated immunity.

Short-duration spaceflight onboard the space shuttle is a unique experience that includes a unique set of stressors that contribute to the dysregulation of immune and endocrine systems).^6, 10^ Astronauts on ISS missions experience many similar stressors for a much longer duration. Recent studies confirmed that astronauts on the ISS continue to experience dysregulation of immune and endocrine systems.^6, 10^ Increased levels of plasma and urinary stress hormones (cortisol, and catecholamines) commonly accompany spaceflight.^11, 12^

Virus reactivation was shown to be associated with the unique combination of stressors associated with spaceflight which include psychosocial stressors of isolation, confinement, anxiety, sleep deprivation, as well as physical exertion, noise, increased radiation, and microgravity.^1, 13^ These stressors may be constant or intermittent. It has been previously suggested that during 4–6 months of spaceflight, astronauts onboard the ISS would acclimate to the unique environment resulting in the recovery of the immune system to normal levels. Therefore, the immune and viral reactivation observations during 10–16 days space shuttle missions may not occur in crewmembers of 4–6 month missions.^6^ Normal immune activity is likely to prevent or mitigate the shedding of the herpes viruses (with exception of EBV) as observed in control subjects. Data in the present study demonstrate that shedding of EBV, CMV, and VZV did not abate during the longer missions onboard the ISS. Rather, virus shedding actually increased in frequency and amplitude (viral copy numbers) of all three viruses tested. As shown in Table 2, VZV shedding increased from 41% in short duration space shuttle to 65% in long duration ISS, EBV increased from 82 to 96%, and CMV increased from 47 to 61% of the crewmembers. Also, shedding for all three viruses persisted throughout the long duration (Early, Mid, and Late) missions. In addition, VZV and CMV shed up to 30 days after longer-duration spaceflight on the ISS unlike for the short-duration spaceflight on the space shuttle where VZV was shed only up to 5 days and CMV shed up to 3 days after flight.^5, 14^ In a recent study, it was reported that immune changes observed in short duration spaceflight actually increased in astronauts after 6 months of ISS flight.^7^

EBV infects approximately 90% of human adults, and 100% of all astronauts (*n* = 86) studied to date were seropositive for EBV.^1, 5, 14^ In this study, all but one of the 23 astronauts studied shed EBV at some time point. VZV is an important health risk to crewmembers (some have experienced shingles before or during the course of spaceflight), and CMV can be immuno-suppressive.^15^ CMV may play a role in immune dysfunction observed in crewmembers. Viral reactivation has been reported in ground-based models of spaceflight although to a lesser extent than spaceflight.^16, 17^ Previously, we have shown that the VZV shed in saliva by crewmembers contains live, infectious virus capable of infecting other crew members.^18, 19^ However, if crewmembers have had exposure to VZV, it is not likely to re-infect but they may spread live infectious virus to immunocompromised or sero-negative individuals with whom they come in contact during or after the flight. Reactivation of VZV can result in shingles or other serious health issues that can compromise mission objectives.

EBV shedding was found in 3% of control subjects while VZV and CMV were found in less than 1%.^1, 5, 14^ Even though the exact mechanism of virus shedding in saliva of astronauts during flight is not fully understood, increased stress and reduced immunity are important contributory factors.^9, 20^ VZV is a neurotropic virus that does not appear in saliva of normal healthy subjects.^21^ However, stressful conditions such as spaceflight can reactivate this virus to replicate in the dorsal root ganglia, and shedding occurs in saliva.^22^

The apparent higher salivary cortisol profile predicted in diurnal cortisol (Fig. 5) is similar to dysregulation associated with psychosocial challenges such as chronic caregiving stress,^23, 24^ early trauma,^25^ and breast cancer.^26^ Herein with longitudinal sampling during the course of extended space flight, there were indications of disruption in cortisol levels reflected by a higher diurnal level at mid-day. Our previous report for short-term space flight suggested that cortisol levels remained unchanged when applying a different metric, area under the curve.^1^ These differences could be due to the impact of longer exposure to microgravity,radiation, work load, and psychological factors operating in longer missions. Furthermore, in the MARS500 investigation,^27^ no relevant shedding was observed during or after isolation, again suggesting the impact of spaceflight (in particular, long-duration spaceflight) as a key contributor to the current findings. The lack of psychological assessments addressing stress levels in crewmembers is a limitation of the present study. Additonally the inability to precisely time each sample collection within a 24-h cycle, limits our capacity to speculate on sources of midday increases. Improved documentation of daily activities on when saliva was collected would contribute significantly to interpreting these observations. However the contraints in collection from rigidly scheduled crewmembers precludes collecting additional information of this nature.

Controlling the reactivation of latent herpes viruses is among the numerous functions of T cells. Percentages of virus-specific T cells have been found to be elevated after short-duration spaceflight.^28^ An investigation of immunity after short duration space shuttle missions found no significant increases in the absolute levels of peptide-specific EBV or CMV-specific CD8^+^ T cells. However, CD8^+^ T-cell subsets, including cytotoxic, central memory, and senescent; T-cell function; and cytokine production profiles were altered during short-duration spaceflight.^6^ The functional capacity of virus-specific T cells during this previous study was in fact decreased.^6^

## Conclusions

Previously, we showed that at least three latent herpes reactivate and shed in astronauts during short-term spaceflight.^1, 5, 14^ Changes in immunity were also observed^6^ and provided a likely mechanistic cause for reactivation of latent viruses.^6, 7^ This study was undertaken to determine if the longer duration missions resulted in a reduction in stress effects and restored viral immunity thus preventing or mitigating the reactivation of these viruses. Unfortunately, in this long duration (6 months) ISS study, immune status did not improve^7^ and consequently, reactivation of EBV, VZV, and CMV actually increased in frequency, duration, and amplitude (viral copy numbers) when compared to values found with short-duration spaceflight. The viruses studied reactivated independently of each other. These findings could impact the design of exploration-class missions during which reactivation of latent viruses could result in an increased risk of wide-ranging adverse medical events.^10^ It may be however, that a partial-gravity environment, e.g., on Mars, would be sufficient to prevent serious viral reactivation. Future research needs to address this question. For now, we conclude that because astronaut’s saliva has significantly increased shedding of VZV DNA during and after spaceflight and this virus has been shown to be infectious in an earlier study,^19^ vaccination of crewmembers with VZV vaccine (zostavax) may be recommended as a countermeasure to the astronauts before they go in space.

## Materials and methods

### Subjects

Twenty-three ISS astronauts (18 male and 5 female; mean age + SE = 53 + 4.9 years) participated in this observational study. These study participants are the same individuals that participated in a recent immune study.^7^ Informed consent was obtained from all subjects who participated in the study and the study was approved by the Institutional Review Board at the Johnson Space Center, Houston, Texas. The nominal mission duration was approximately 180 days. Two crewmembers participated in shorter missions of approximately 2–3 months. For those crewmembers; only two samplings occurred during flight, with data aligned with the ‘early’ and ‘mid’ for the 6-month crewmembers.

Twenty apparently healthy age—and gender matched subjects (16 males and 4 females, mean age ± SE of 49.3 ± 4.9 years) participated as ground-based viral reactivation controls. With the exception of the diurnal saliva collections, the control subjects were sampled and processed at the same time for the same assays as the ISS crewmembers. All astronauts and control subjects were EBV, CMV, VZV, and HSV-1 seropositive as tested by measuring IgG with an ELISA based commercial assay at baseline (180 days before flight) assessment. A total of 1044 samples from 23 astronauts were collected before, during and after long-duration spaceflight on the ISS and were analyzed upon return to Earth. Saliva accounted for 644 samples, 207 samples were urine, and 193 were blood samples. Saliva samples were assayed for EBV, VZV, and HSV-1/2 and HHV6; urine samples were analyzed for CMV. This study was observational and exploratory, making use of all data from participating astronauts, and as such was not designed to achieve any particular level of power for detecting pre-specified effects, nor could this work be replicated in a laboratory setting.

### Sample collection

Saliva samples for viral assessments were collected using Salivette cotton rolls (Sarstedt, Inc., Newton, NC immediately after their sleep cycle, before eating and brushing their teeth. To collect a sample, the subject placed the cotton roll in their mouth until it became saturated with saliva (2–3 min). The saturated salivette was then placed in a Ziploc^®^ bag containing 1 mL of stability buffer (0.5% SDS, 10 mM Tris-Cl, and 1 mM EDTA. The samples were stored at ambient temperature for approximately 2 weeks before return to Earth for analysis.^1^

Four saliva samples were collected at each of 7 sampling sessions, approximately corresponding to the following time points: Sessions 1–2: 180 and 45 days before flight; Sessions 3–5: during flight at mission day 14 days (early), between mission days 60–120 days (mid-mission) and about 180 days (late); Sessions 6–7: 3 h after landing and 30 days after landing (Fig. 1). A 10 mL EDTA anti coagulated peripheral blood sample and a 24-h urine pool were also collected before and after each mission time point. Only one blood sample was taken at each inflight time point, as shown in Table 3.

**Table 3:** Sample schedule for long-duration spaceflight

	Number of samples						
	Before flight		During flight			After flight	
	180 days	45 days	Early mission (14 days)	Mid mission (2–4 months)	Late mission (5–6 months)	Landing day	30 days after
Saliva Liquid*	4	4	4	4	4	4	4
Diurnal saliva	1	1	1	1	1	1	1
Urine	1	1	0	1	0	1	1
Blood	1	1	1	1	1	1	1

* About 2 mL saliva was collected every other day around sampling period, e.g., 180 days; diurnal saliva samples collected 5 times a day on filter paper a 10 mL EDTA blood and a 24 h urine pool were collected around the above identified time points

Samples for control subjects (four saliva samples, a urine sample from a 24-h pool, and a blood sample (10 mL, EDTA) were collected at corresponding time points for a 6—month simulated spaceflight mission. Control samples were treated the same way as astronauts’ samples and processed in batches after each subject completed the simulated spaceflight schedule.

Urine samples from the participating crewmembers were obtained via a sample sharing agreement with another NASA investigation. Twenty four-hour urine pool samples were collected before and after flight. Urine voids during flight contained 1 mL of a LiCl solution as a volume marker. The 24-h urine pool was thoroughly mixed, and a syringe aliquot was obtained and then frozen until return to Earth for analyses about 6–12 months later. After landing, the flight urine samples were analyzed for lithium concentration to determine void volume and subsequently to prepare 24-h pools, as previously described.^29, 30^

### Sample processing and storage

Upon return to Earth, flight saliva samples collected via Salivettes were centrifuged to separate fluid from the cotton, and the supernatant fluid was stored frozen (−70 °C) until processed. Ground-based analysis verified that the stability buffer preserved viral DNA in saliva for subsequent polymerase chain reaction (PCR) analysis for up to 240 days^14^ Ten-fold concentration of saliva was achieved by centrifugation using a Microsep concentrator 100kD (Pall Filtron Corp., Northborough, MA). For CMV, urine (3 mL) was concentrated to ~200 µL using a 100 kD filtration unit as mentioned above.

Plasma was separated by centrifugation and stored at −70 °C until processed. All samples collected from each subject were processed together to avoid inter-assay variations between subjects or assay inconsistencies between laboratories.

### Detection of viral DNA

Viral DNA was extracted by a nonorganic extraction method (Qiagen Inc., Chatsworth, CA) QIAamp Viral RNA Kits (Qiagen Inc., Santa Clarita, CA) were used to extract viral genomic DNA from concentrated saliva and urine, and each assay was performed according to manufacturer’s instructions. HSV1, HSV2, EBV, HHV6, and VZV DNA were measured in saliva and CMV DNA in urine by real-time PCR using an ABI 7900 (Applied Biosystems, Foster City, CA) PCR system. The primers and probes used for EBV, VZV, and CMV have been published previously.^1^

### Measurement of antibody titer

Anti-viral antibody titers were determined by indirect immunofluorescence as previously described.^31^ Commercially prepared substrate slides and control sera (Bion Enterprises, Park Ridge, IL) were used for determining IgG antibody titers to EBV viral capsid antigen (VCA) and early antigen, EBV-nuclear antigen, VZV, and CMV as described.^1^

### Diurnal salivary cortisol and DHEA

Diurnal dry saliva samples (5 per sampling day) were collected at awakening and 30 min, 6 h, and 10 h after awakening, as well as on retiring, using a unique filter-paper collection method. In short, the subject wet the filter paper with saliva, which was then air-dried at room temperature storage until return to Earth. All of a subject’s samples were assayed on the same plate. Filters were processed for cortisol and DHEA in a similar manner as previously described.^32, 33^ Intra-assay and inter-assay coefficients of variation for cortisol and DHEA were less than 5 and 10%, respectively, using this procedure.

### Measurement of plasma cortisol

Stored plasma samples were thawed, and cortisol was measured by EIA using commercially available kits (Alpco Diagnostics, Salem, NH). Samples were batch analyzed to minimize inter-assay variation.

### Statistical analysis

#### Viral reactivation

For each subject, EBV and VZV maximum copy numbers within the four replicate saliva samples at each of 7 time points were analyzed to (a) compare the effect of mission time points on all copy numbers (including zeroes for cases of no shedding) and (b) to compare means of log-transformed copy numbers for those samples in which a virus was shed. Single values of CMV copies were similarly analyzed as is. For (a) above we used Friedman’s rank-based ANOVA^34^ with follow-up multiple comparisons if the overall test for equal mean ranks was rejected (two-sided *p* < 0.05). No analysis was made on data from HSV1, HSV2, and HHV6 since these viruses were not shed at any time point. For (b) we used mixed model linear regression analysis^35^ with Sidak-adjusted *P*-values for post-hoc comparisons of each time point to the previous one.

#### Antibody titers

By nature, antibody titers are discrete, taking only relatively few distinct values. Therefore, we compared the antibody titers between time points with a repeated-measures version of an ordered logit regression model,^36^ which is designed to analyze ordered categorical data.

#### EBV-DNA copies

This data was highly variable, with a considerable number of observations below detectable limits (2 copies), while others ranged over several orders of magnitude. We analyzed this outcome using median regression^37^ with samples below detection limits being treated as having produced 2 copies.

#### Plasma cortisol

Median plasma cortisol levels at the seven time points were compared using median regression with cluster bootstrapped samples at the subject level. The presence of a few outliers, which were verified to be valid, precluded the use of mixed-model regression for this purpose.

#### Hormone data (saliva)

The main outcomes here were cortisol concentration (μg/dl) and DHEA concentration (pg/mL) measured at various hours within a day, starting at awakening. The goal of the analysis was so see if the mean daily trajectories of these outcomes differed by flight period or phase. To do this we constructed random-effects regression models^35^ (one for each mission time point) for the mean daily trajectories of each outcome on a log scale using a 3-knot cubic spline, then compared the parameters of the regression model between pre-flight and each of the other phases. Comparisons were assessed in terms of two-sided *P*-values with Bonferroni adjustment when appropriate. Model assumptions were checked with Q–Q plots of residuals. Cortisol data collected within 1 h after awakening was not used in any of the analyses because this value represents a different underlying control system^38^ as well as the difficulty of modeling the high variability associated with the rise in cortisol concentration at that time. A detailed description of these types of analyses as well as an example of one such analysis is given in supplementary information files.

#### Code availability

All statistical analyses were performed with user-written sequences of commands in Stata 14 software. Some of these commands may be seen preceding the analysis results in the supplementary material.

## Electronic supplementary material

Appendix

Supplementary Information
