# Supplementary material for: Latent virus reactivation in astronauts on the international space station
Source: NPJ Microgravity. 2017 Apr 12;3:11. doi: 10.1038/s41526-017-0015-y (PMC5445581; doi:10.1038/s41526-017-0015-y)
Supplement: Supplementary file 1 — Appendix [file 41526_2017_15_MOESM1_ESM.docx]

**Appendix –Regression Analysis Models for Hormone Data**

*Cubic Splines*

Let *y* denote the log-transformed outcome (cortisol or DHEA). For each period in an analysis, the original time points (*t*) were augmented by 101 equally spaced values of *t* from 0 to 20 hours, in steps of 0.2 hours. The mean trajectory $E\left( y|t \right)$ was then modeled as a linear combination of two basis functions $U_{1}\left( t \right)$ and $U_{2}\left( t \right)$, where $U_{1}\left( t \right)=t$, and $U_{2}\left( t \right)$ is a restricted cubic spline constructed with knots at the10th, 50th, and 90th percentiles of all *t*-values [ref Harrell].

*Nomenclature:*

Although actual days of in-flight sample collection differed by subject, these days were grouped into three intervals, which we refer to as “periods”: early flight, mid-flight, and late flight. We also use the term “phase” to distinguish between samples gathered pre-, in, or post-flight, regardless of the actual collection day.

*Analysis Model 1. Comparing daily trajectories between the three flight periods: early (k = 1), mid (k = 2), late (k = 3) and with pre-flight (k = 0).*

Let $y_{ikj}$ denote the log-transformed hormone concentration for the *i*-th subject as measured from the *j*-th sample collection at time $t=t_{ikj}$ after awakening during the *k*-th flight period ( *j* = 1, 2, . . , *N_ik_*) . Then the mixed model for $y_{ikj}$ is given by

pre-flight (*k* = 0):

$$y_{i0j}= \mu+\beta_{1}U_{1}\left( t_{i0j} \right)+\beta_{2}U_{2}\left( t_{i0j} \right)+u_{i}+e_{i0j}$$

in-flight (*k* = 1, 2, 3):

$$y_{ikj}= \mu+\alpha_{k}+\beta_{1}U_{1}\left( t_{ikj} \right)+\beta_{2}U_{2}\left( t_{ikj} \right)+\left( \alpha\beta\right)_{k1}I_{k}U_{1}\left( t_{ikj} \right)+\left( \alpha\beta\right)_{k2}I_{k}U_{2}\left( t_{ikj} \right)+u_{i}+z_{i}+e_{ikj}$$

Parameters in this model include:

Fixed coefficients $\mu, \alpha_{k},\beta_{1}, \beta_{2},\left( \alpha\beta\right)_{k1}\left( \alpha\beta\right)_{k2}$. Here $\left( \alpha\beta\right)_{k1}$and $\left( \alpha\beta\right)_{k2}$ are differential fixed effects of the *k*-th in-flight period on $U_{1}\left( t \right)$ and $U_{2}\left( t \right)$, respectively.

Random effects

1. $u_{i}\sim N(0,\sigma_{u}^{2})$ - an overall random contribution to the intercept for the *i*-th subject.
2. $z_{i}\sim N(0,\sigma_{v}^{2})$ - a random perturbation to $u_{i}$ for all in-flight periods. This represents a random interaction between subject and flight phase (pre-flight or in-flight).
3. $e_{ikj}\sim N(0,\sigma^{2})$ - a random within-subject error term .

All random effects are modeled as mutually independent.

This model was used to decide whether there was enough information in the data to permit separate comparisons of each in-flight period with pre-flight, or whether the data from the in-flight periods should be combined and tested in aggregate against the pre-flight period.

*Analysis Model 2. Comparing daily trajectories between the in-flight phase (φ =1) and the pre-flight phase (φ = 0).*

Model 2 was used for analysis if the results of applying Model 1 were consistent with the assumption that all three in-flight periods elicit the same response. This model has the same form as Model 1, except that the index *k* (for period) is replaced by the index *φ* (for phase) and the latter takes on only two values: 0 and 1. Here $y_{i\varphi j}$ denotes the *j*-th preflight sample if *φ* = 0, and the *j*-th in-flight sample if *φ* = 1, where for in-flight samples, $j=1, 2, ..,\sum_{k=1}^{3} N_{ik}$.

*Analysis Model 3. Comparing daily trajectories between either recovery period; early (k =4), or late (k = 5), and the pre-flight period (k = 0).*

This model was fit separately to compare trajectories for early recovery vs. pre-flight, and also for late recovery vs. pre-flight. Model 3 has the same form as Model 1, except that the values of *k* are now 0 (pre-flight), and either 4 or 5 (post-flight).

All models were fit using the method of restricted maximum likelihood, which has been shown to provide more accurate inference than maximum-likelihood when sample sizes are small Inference on the effect of flight or recovery relative to pre-flight on daily trajectories was made using Wald tests.

*Example*

As an example, Table 1 show the Stata output after fitting Model 2. Figures 1 and 2 show the estimated mean daily trajectory for log cortisol concentration for pre-flight and in-flight samples, respectively. Superimposed are the original data (gray) and adjusted data, which is the original data with the best-linear-unbiased (B.L.U.P.) predicted values of the subject-level random effects removed (solid dots).

Table 1. Stata Results after Fitting Model 2.

Mixed-effects REML regression Number of obs = 340

Group variable: isub Number of groups = 21

Obs per group:

min = 4

avg = 16.2

max = 41

Wald chi2(5) = 64.79

Log restricted-likelihood = -455.97285 Prob > chi2 = 0.0000

------------------------------------------------------------------------------

zyc | Coef. Std. Err. z P>|z| [95% Conf. Interval]

-------------+----------------------------------------------------------------

phase |

In-flight | -.1753754 .2519939 -0.70 0.486 -.6692745 .3185236

U1 | -.1434237 .028458 -5.04 0.000 -.1992003 -.0876471

|

phase#c.U1 |

In-flight | .1022072 .0355508 2.87 0.004 .0325288 .1718855

|

U2 | .09419 .0352824 2.67 0.008 .0250378 .1633422

|

phase#c.U2 |

In-flight | -.0913781 .0440361 -2.08 0.038 -.1776873 -.0050688

|

_cons | 1.39732 .1789363 7.81 0.000 1.046611 1.748029

------------------------------------------------------------------------------

------------------------------------------------------------------------------

Random-effects Parameters | Estimate Std. Err. [95% Conf. Interval]

-----------------------------+------------------------------------------------

isub: Independent |

sd(phase) | .8045723 .1586003 .5467303 1.184015

sd(_cons) | .5020963 .1180301 .3167315 .7959446

-----------------------------+------------------------------------------------

sd(Residual) | .8049213 .0327152 .7432882 .8716651

------------------------------------------------------------------------------

LR test vs. linear model: chi2(2) = 214.15 Prob > chi2 = 0.0000

Note: LR test is conservative and provided only for reference.

Test For Overall Phase Effect (in-flight vs pre-flight)

( 1) [zyc]1.phase = 0

( 2) [zyc]1.phase#c.U1 = 0

( 3) [zyc]1.phase#c.U2 = 0

chi2( 3) = 11.39

Prob > chi2 = 0.0098

Figure1. Estimated Mean Daily Trajectory for Pre-flight Samples

Figure 2. Estimated Mean Daily Trajectory for In-flight Samples.

Figures 3-5 show q-q plots of the three types of residuals corresponding to B.L.U.P. values of $e_{ikj}$, $u_{i}$, and $z_{i}$, respectively. It can be seen that normality assumptions for $e_{ikj}$ and $u_{i}$ are quite good, however the assumption of normality of $z_{i}$ is not well satisfied because of one outlier subject (“X”). Inclusion of this subject biases the estimate towards zero and inflates the standard error of the in-flight main effect coefficient and thus reduces the power to detect a phase effect. Without this subject in the analysis, the test for an overall phase effect produces a chi-squared value of 15.4 (p = 0.0015), as compared with 11.4 (p = 0.0098) with the subject included.

Figure 3. q-q plot for Lowest-level Residuals ($e_{ikj}$).

Figure 4. q-q plot for Subject-level Random Intercepts ($u_{i}$).

Figure 5. q-q plot for Subject-level Random Interactions ($z_{i}$).

*Reference*

Diggle, P., Liang, K. Y. and Zeger, S. L. 1995. *Analysis of Longitudinal Data.* Oxford Science Publications, Clarendon Press: Oxford. pp.64-68.

(Diggle, P., Liang, K. Y. and Zeger, S. L. 1995. *Analysis of Longitudinal Data.* Oxford Science Publications, Clarendon Press: Oxford. pp.68-77.(robust estimation of SE) pp.64-68 (REML).
