# Supplementary material for: Latent virus reactivation in astronauts on the international space station
Source: NPJ Microgravity. 2017 Apr 12;3:11. doi: 10.1038/s41526-017-0015-y (PMC5445581; doi:10.1038/s41526-017-0015-y)
Supplement: Supplementary file 2 — Supplementary Information [file 41526_2017_15_MOESM2_ESM.docx]

.

***Viral Reactivation*:**

Compare Mission time points with respect to

EBV (based on ebv3_anal.do)

**Raw counts of shedding instances**

use ebv_long_031416,clear

count if y > 0 & y < . 43

count if y == 0 & y < . 118

di 7*23 161 (23 subjects x 7 time periods)

**a)Friedman’s Test on all copy numbers (including zeros)**

use ebv_long_031416,clear

drop z phase nz

reshape wide y,i(isub) j(tk)

save temp,replace

noi fried y*

Friedman's Two-way Rank Test - 23 observations, 7 variables

---------------------------------------------------------------

S' = 11.90 P = 0.06421

Multiple Comparisons (not shown - non signficant)

**b)log-transformations of copy numbers given shedding**

. use "C:\Users\afeiveso\Documents\stfiles\ebv_long_031416.dta",clear

. mixed z i.tk ||isub: ,nolog reml

Mixed-effects REML regression Number of obs = 43

Group variable: isub Number of groups = 20

Obs per group:

min = 1

avg = 2.1

max = 3

Wald chi2(6) = 112.94

Log restricted-likelihood = -7.4624608 Prob > chi2 = 0.0000

------------------------------------------------------------------------------

z | Coef. Std. Err. z P>|z| [95% Conf. Interval]

-------------+----------------------------------------------------------------

tk |

L-45 | -.0711427 .1635534 -0.43 0.664 -.3917015 .2494162

Early | .5548467 .2263203 2.45 0.014 .1112671 .9984263

Mid | .8556983 .1720436 4.97 0.000 .5184991 1.192898

Late | .8439604 .1629585 5.18 0.000 .5245675 1.163353

R+0 | .1632843 .1627305 1.00 0.316 -.1556617 .4822303

R+30 | .3048401 .1765513 1.73 0.084 -.0411941 .6508743

|

_cons | 1.945399 .1438253 13.53 0.000 1.663506 2.227291

------------------------------------------------------------------------------

------------------------------------------------------------------------------

Random-effects Parameters | Estimate Std. Err. [95% Conf. Interval]

-----------------------------+------------------------------------------------

isub: Identity |

var(_cons) | .0159362 .0173111 .0018956 .1339746

-----------------------------+------------------------------------------------

var(Residual) | .0502679 .0172145 .0256917 .0983534

------------------------------------------------------------------------------

LR test vs. linear model: chibar2(01) = 1.06 Prob >= chibar2 = 0.1515

. contrast r.tk,mcompare(sidak)

Contrasts of marginal linear predictions

Margins : asbalanced

----------------------------------------------------------------

| Sidak

| df chi2 P>chi2 P>chi2

------------------+---------------------------------------------

z |

tk |

(L-45 vs L-180) | 1 0.19 0.6636 0.9986

(Early vs L-180) | 1 6.01 0.0142 0.0824

(Mid vs L-180) | 1 24.74 0.0000 0.0000

(Late vs L-180) | 1 26.82 0.0000 0.0000

(R+0 vs L-180) | 1 1.01 0.3157 0.8973

(R+30 vs L-180) | 1 2.98 0.0842 0.4102

Joint | 6 112.94 0.0000

----------------------------------------------------------------

Note: Sidak-adjusted p-values are reported for tests on

individual contrasts only.

---------------------------

| Number of

| Comparisons

-------------+-------------

z |

tk | 6

---------------------------

-------------------------------------------------------------------

| Sidak

| Contrast Std. Err. [95% Conf. Interval]

------------------+------------------------------------------------

z |

tk |

(L-45 vs L-180) | -.0711427 .1635534 -.501458 .3591727

(Early vs L-180) | .5548467 .2263203 -.0406107 1.150304

(Mid vs L-180) | .8556983 .1720436 .403045 1.308352

(Late vs L-180) | .8439604 .1629585 .4152103 1.27271

(R+0 vs L-180) | .1632843 .1627305 -.264866 .5914346

(R+30 vs L-180) | .3048401 .1765513 -.1596731 .7693533

-------------------------------------------------------------------

.To compare with R+30

. mixed z b7.tk ||isub: ,nolog reml

Mixed-effects REML regression Number of obs = 43

Group variable: isub Number of groups = 20

Obs per group:

min = 1

avg = 2.1

max = 3

Wald chi2(6) = 112.94

Log restricted-likelihood = -7.4624608 Prob > chi2 = 0.0000

------------------------------------------------------------------------------

z | Coef. Std. Err. z P>|z| [95% Conf. Interval]

-------------+----------------------------------------------------------------

tk |

L-180 | -.3048401 .1765513 -1.73 0.084 -.6508743 .041194

L-45 | -.3759828 .1305321 -2.88 0.004 -.631821 -.1201446

Early | .2500066 .2005335 1.25 0.213 -.1430318 .6430449

Mid | .5508582 .1549672 3.55 0.000 .2471281 .8545883

Late | .5391203 .1324698 4.07 0.000 .2794841 .7987564

R+0 | -.1415558 .137936 -1.03 0.305 -.4119055 .1287938

|

_cons | 2.250239 .1116095 20.16 0.000 2.031488 2.46899

------------------------------------------------------------------------------

------------------------------------------------------------------------------

Random-effects Parameters | Estimate Std. Err. [95% Conf. Interval]

-----------------------------+------------------------------------------------

isub: Identity |

var(_cons) | .0159362 .0173111 .0018956 .1339745

-----------------------------+------------------------------------------------

var(Residual) | .0502679 .0172145 .0256917 .0983534

------------------------------------------------------------------------------

LR test vs. linear model: chibar2(01) = 1.06 Prob >= chibar2 = 0.1515

. contrast r.tk,mcompare(sidak)

Contrasts of marginal linear predictions

Margins : asbalanced

---------------------------------------------------------------

| Sidak

| df chi2 P>chi2 P>chi2

-----------------+---------------------------------------------

z |

tk |

(L-180 vs R+30) | 1 2.98 0.0842 0.4102

(L-45 vs R+30) | 1 8.30 0.0040 0.0236

(Early vs R+30) | 1 1.55 0.2125 0.7615

(Mid vs R+30) | 1 12.64 0.0004 0.0023

(Late vs R+30) | 1 16.56 0.0000 0.0003

(R+0 vs R+30) | 1 1.05 0.3048 0.8871

Joint | 6 112.94 0.0000

---------------------------------------------------------------

Note: Sidak-adjusted p-values are reported for tests on

individual contrasts only.

---------------------------

| Number of

| Comparisons

-------------+-------------

z |

tk | 6

---------------------------

VZV (based on vzv3_anal.do)

use vzv_long_031416,clear

drop zm iy n phase

reshape wide ym,i(isub) j(tk)

save temp,replace

fried y*

. fried y*

Friedman's Two-way Rank Test - 23 observations, 7 variables

---------------------------------------------------------------

S' = 42.30 P = 0.00000

Multiple Comparisons

------------------------------------------------------

Var1 Var2 Ru - Rv P (2-sided)

ym1 ym2 0.00 1.0000

ym1 ym3 -27.50 0.4957

ym1 ym4 -32.00 0.3041

ym1 ym5 -50.00 0.0115

ym1 ym6 -36.00 0.1752

ym1 ym7 -5.00 0.9999

ym2 ym3 -27.50 0.4957

ym2 ym4 -32.00 0.3041

ym2 ym5 -50.00 0.0115

ym2 ym6 -36.00 0.1752

ym2 ym7 -5.00 0.9999

ym3 ym4 -4.50 0.9999

ym3 ym5 -22.50 0.7232

ym3 ym6 -8.50 0.9974

ym3 ym7 22.50 0.7232

ym4 ym5 -18.00 0.8832

ym4 ym6 -4.00 1.0000

ym4 ym7 27.00 0.5188

ym5 ym6 14.00 0.9632

ym5 ym7 45.00 0.0347

ym6 ym7 31.00 0.3431

Drop pre-flight time periods with no shedding and compare the rest

. fried ym3-ym7

Friedman's Two-way Rank Test - 23 observations, 5 variables

---------------------------------------------------------------

S' = 17.30 P = 0.00169

Multiple Comparisons

------------------------------------------------------

Var1 Var2 Ru - Rv P (2-sided)

ym3 ym4 -5.50 0.9861

ym3 ym5 -18.50 0.4184

ym3 ym6 -6.50 0.9742

ym3 ym7 15.50 0.5982

ym4 ym5 -13.00 0.7443

ym4 ym6 -1.00 1.0000

ym4 ym7 21.00 0.2867

ym5 ym6 12.00 0.7966

ym5 ym7 34.00 0.0132

ym6 ym7 22.00 0.2415

**b)log-transformations of VZV copy numbers given shedding**

**[Note: Shedding occurred in only 5 of the 7 time points (none pre-flight)]**

. use vzv_long_031416,clear

. mixed zm i.tk if tk>2||isub: ,nolog reml

Mixed-effects REML regression Number of obs = 43

Group variable: isub Number of groups = 17

Obs per group:

min = 1

avg = 2.5

max = 4

Wald chi2(4) = 9.18

Log restricted-likelihood = -16.731226 Prob > chi2 = 0.0567

------------------------------------------------------------------------------

zm | Coef. Std. Err. z P>|z| [95% Conf. Interval]

-------------+----------------------------------------------------------------

tk |

Mid | .2585443 .1602432 1.61 0.107 -.0555266 .5726153

Late | .3716247 .1430013 2.60 0.009 .0913473 .651902

R+0 | .0571975 .1482241 0.39 0.700 -.2333165 .3477114

R+30 | .0952974 .2577992 0.37 0.712 -.4099798 .6005746

|

_cons | 2.195609 .109926 19.97 0.000 1.980158 2.41106

------------------------------------------------------------------------------

------------------------------------------------------------------------------

Random-effects Parameters | Estimate Std. Err. [95% Conf. Interval]

-----------------------------+------------------------------------------------

isub: Identity |

var(_cons) | 1.33e-23 1.65e-22 3.10e-34 5.66e-13

-----------------------------+------------------------------------------------

var(Residual) | .1087534 .0249498 .0693687 .1704992

------------------------------------------------------------------------------

LR test vs. linear model: chibar2(01) = 0.00 Prob >= chibar2 = 1.0000

. contrast r.tk,mcompare(sidak)

Contrasts of marginal linear predictions

Margins : asbalanced

---------------------------------------------------------------

| Sidak

| df chi2 P>chi2 P>chi2

-----------------+---------------------------------------------

zm |

tk |

(Mid vs Early) | 1 2.60 0.1066 0.3631

(Late vs Early) | 1 6.75 0.0094 0.0369

(R+0 vs Early) | 1 0.15 0.6996 0.9919

(R+30 vs Early) | 1 0.14 0.7116 0.9931

Joint | 4 9.18 0.0567

---------------------------------------------------------------

Note: Sidak-adjusted p-values are reported for tests on

individual contrasts only.

---------------------------

| Number of

| Comparisons

-------------+-------------

zm |

tk | 4

---------------------------

------------------------------------------------------------------

| Sidak

| Contrast Std. Err. [95% Conf. Interval]

-----------------+------------------------------------------------

zm |

tk |

(Mid vs Early) | .2585443 .1602432 -.140608 .6576967

(Late vs Early) | .3716247 .1430013 .0154206 .7278287

(R+0 vs Early) | .0571975 .1482241 -.3120163 .4264112

(R+30 vs Early) | .0952974 .2577992 -.5468586 .7374534

------------------------------------------------------------------

.

CMV (based on cmv3_anal.do)

[Note: CMV samples were obtained only for two pre, one in, and two post (5 total)]

use cmv_long_032416,clear

drop zm phase orm

reshape wide y,i(isub) j(period)

save temp,replace

Friedman's Two-way Rank Test - 23 observations, 5 variables

---------------------------------------------------------------

S' = 26.55 P = 0.00002

Multiple Comparisons

------------------------------------------------------

Var1 Var2 Ru - Rv P (2-sided)

y1 y2 -15.50 0.5982

y1 y3 -34.00 0.0132

y1 y4 -14.00 0.6878

y1 y5 -4.00 0.9959

y2 y3 -18.50 0.4184

y2 y4 1.50 0.9999

y2 y5 11.50 0.8208

y3 y4 20.00 0.3364

y3 y5 30.00 0.0412

y4 y5 10.00 0.8843

Drop L-180 time period with no shedding and compare the other four:

. fried y2-y5

Friedman's Two-way Rank Test - 23 observations, 4 variables

---------------------------------------------------------------

S' = 16.73 P = 0.00080

Multiple Comparisons

------------------------------------------------------

Var1 Var2 Ru - Rv P (2-sided)

y2 y3 -16.00 0.2604

y2 y4 1.00 0.9995

y2 y5 9.00 0.7331

y3 y4 17.00 0.2107

y3 y5 25.00 0.0223

y4 y5 8.00 0.7976

**b)log-transformations of CMV copy numbers given shedding**

**[Note: Shedding occurred in only 4 of the 5 time points (none at L-180)]**

. use cmv_long_032416,clear

. mixed zm i.period if period>1||isub: ,nolog reml

Mixed-effects REML regression Number of obs = 27

Group variable: isub Number of groups = 15

Obs per group:

min = 1

avg = 1.8

max = 3

Wald chi2(3) = 16.29

Log restricted-likelihood = -10.742682 Prob > chi2 = 0.0010

------------------------------------------------------------------------------

zm | Coef. Std. Err. z P>|z| [95% Conf. Interval]

-------------+----------------------------------------------------------------

period |

3 | .4789173 .1299857 3.68 0.000 .2241499 .7336846

4 | .0863925 .1466418 0.59 0.556 -.2010202 .3738052

5 | .145157 .2404023 0.60 0.546 -.3260228 .6163367

|

_cons | 1.998853 .1241708 16.10 0.000 1.755483 2.242223

------------------------------------------------------------------------------

------------------------------------------------------------------------------

Random-effects Parameters | Estimate Std. Err. [95% Conf. Interval]

-----------------------------+------------------------------------------------

isub: Identity |

var(_cons) | .0700703 .0434727 .02077 .2363919

-----------------------------+------------------------------------------------

var(Residual) | .0592072 .026187 .0248825 .1408823

------------------------------------------------------------------------------

LR test vs. linear model: chibar2(01) = 3.79 Prob >= chibar2 = 0.0258

. contrast r.period,mcompare(sidak)

Contrasts of marginal linear predictions

Margins : asbalanced

-----------------------------------------------------------

| Sidak

| df chi2 P>chi2 P>chi2

-------------+---------------------------------------------

zm |

period |

(3 vs 2) | 1 13.57 0.0002 0.0007

(4 vs 2) | 1 0.35 0.5558 0.9123

(5 vs 2) | 1 0.36 0.5460 0.9064

Joint | 3 16.29 0.0010

-----------------------------------------------------------

Note: Sidak-adjusted p-values are reported for tests on

individual contrasts only.

---------------------------

| Number of

| Comparisons

-------------+-------------

zm |

period | 3

---------------------------

--------------------------------------------------------------

| Sidak

| Contrast Std. Err. [95% Conf. Interval]

-------------+------------------------------------------------

zm |

period |

(3 vs 2) | .4789173 .1299857 .1685454 .7892891

(4 vs 2) | .0863925 .1466418 -.2637497 .4365348

(5 vs 2) | .145157 .2404023 -.4288606 .7191746

--------------------------------------------------------------

**EBV DNA Levels in PBMC’s**

. use EBV_DNA_PBMCdata_long.dta,clear

(Ray Stowe's EBV DNA PBMC data)

.

. gen z10y=log10(y)

(12 missing values generated)

.

. bootstrap _b,reps(100) cluster(isub):qreg z10y i.tk

(running qreg on estimation sample)

Bootstrap replications (100)

----+--- 1 ---+--- 2 ---+--- 3 ---+--- 4 ---+--- 5

.................................................. 50

...............................x.................. 100

Median regression Number of obs = 135

Raw sum of deviations 73.6058 (about .90309)

Min sum of deviations 69.23461 Pseudo R2 = 0.0594

(Replications based on 21 clusters in isub)

------------------------------------------------------------------------------

| Observed Bootstrap Normal-based

z10y | Coef. Std. Err. z P>|z| [95% Conf. Interval]

-------------+----------------------------------------------------------------

tk |

2 | -.7403627 .7124368 -1.04 0.299 -2.136713 .6559879

3 | -.7403627 .7098599 -1.04 0.297 -2.131663 .6509372

4 | .7579479 .8758136 0.87 0.387 -.9586152 2.474511

5 | -.0413927 1.093738 -0.04 0.970 -2.18508 2.102294

6 | .3565474 .9989947 0.36 0.721 -1.601446 2.314541

7 | .1047354 .8543299 0.12 0.902 -1.56972 1.779191

|

_cons | 1.041393 .7134948 1.46 0.144 -.3570315 2.439817

------------------------------------------------------------------------------

. test 2.tk=3.tk=4.tk=5.tk=6.tk=7.tk=0

( 1) 2.tk - 3.tk = 0

( 2) 2.tk - 4.tk = 0

( 3) 2.tk - 5.tk = 0

( 4) 2.tk - 6.tk = 0

( 5) 2.tk - 7.tk = 0

( 6) 2.tk = 0

chi2( 6) = 8.30

Prob > chi2 = 0.2167

**PLASMA CORTISOL**

. use "C:\Users\afeiveso\Documents\stfiles\plasma_cort_iss_long.dta"

. set seed 7777777

. bootstrap _b,reps(1000) nodots cluster(isub):qreg y i.sess

Median regression Number of obs = 136

Raw sum of deviations 777.105 (about 20.370001)

Min sum of deviations 749.97 Pseudo R2 = 0.0349

(Replications based on 21 clusters in isub)

------------------------------------------------------------------------------

| Observed Bootstrap Normal-based

y | Coef. Std. Err. z P>|z| [95% Conf. Interval]

-------------+----------------------------------------------------------------

sess |

L-45 | 1.060001 4.222317 0.25 0.802 -7.215589 9.335591

Early | 1.730001 5.526538 0.31 0.754 -9.101813 12.56182

Mid | -4 3.686384 -1.09 0.278 -11.22518 3.22518

Late | -4.91 3.995477 -1.23 0.219 -12.74099 2.920992

R+0 | 2.83 5.414378 0.52 0.601 -7.781987 13.44199

R+30 | 4.720001 4.911964 0.96 0.337 -4.907272 14.34727

|

_cons | 20.06 3.061417 6.55 0.000 14.05973 26.06027

------------------------------------------------------------------------------

. test 2.sess=3.sess=4.sess=5.sess=6.sess=7.sess=0

( 1) 2.sess - 3.sess = 0

( 2) 2.sess - 4.sess = 0

( 3) 2.sess - 5.sess = 0

( 4) 2.sess - 6.sess = 0

( 5) 2.sess - 7.sess = 0

( 6) 2.sess = 0

chi2( 6) = 11.07

Prob > chi2 = 0.0863

**EBV Anti-viral Antibody Titers**

. use "C:\Users\afeiveso\Documents\stfiles\abEBV_VCAdata_long.dta"

(Ray Stowe's EBV antibody data)

. xtset isub

panel variable: isub (balanced)

. xtologit y i.tk,i(isub) nolog

Random-effects ordered logistic regression Number of obs = 136

Group variable: isub Number of groups = 21

Random effects u_i ~ Gaussian Obs per group:

min = 6

avg = 6.5

max = 7

Integration method: mvaghermite Integration pts. = 12

Wald chi2(6) = 5.13

Log likelihood = -148.46157 Prob > chi2 = 0.5268

------------------------------------------------------------------------------

y | Coef. Std. Err. z P>|z| [95% Conf. Interval]

-------------+----------------------------------------------------------------

|

tk |

2 | .8105135 .6704802 1.21 0.227 -.5036036 2.124631

3 | .5199543 .7787146 0.67 0.504 -1.006298 2.046207

4 | 1.189165 .6832246 1.74 0.082 -.149931 2.52826

5 | .7887068 .6838781 1.15 0.249 -.5516696 2.129083

6 | 1.109977 .6775858 1.64 0.101 -.2180671 2.43802

7 | 1.332782 .6780929 1.97 0.049 .0037446 2.66182

-------------+----------------------------------------------------------------

/cut1 | -6.875464 1.350258 -5.09 0.000 -9.521921 -4.229008

/cut2 | -5.40047 1.144839 -4.72 0.000 -7.644312 -3.156627

/cut3 | -1.832627 1.000741 -1.83 0.067 -3.794044 .128789

/cut4 | -.1038907 .9792986 -0.11 0.916 -2.023281 1.815499

/cut5 | 4.550047 1.078375 4.22 0.000 2.436471 6.663623

/cut6 | 9.581562 1.793588 5.34 0.000 6.066194 13.09693

-------------+----------------------------------------------------------------

/sigma2_u | 13.44748 5.220755 6.283133 28.78097

------------------------------------------------------------------------------

LR test vs. ologit model: chibar2(01) = 122.93 Prob >= chibar2 = 0.0000

Also see abdata_anomaly.docx

**CMV Anti-viral Antibody Titers**

. use "C:\Users\afeiveso\Documents\stfiles\abCMV_VCAdata_long.dta"

(Ray Stowe's EBV antibody data)

. xtset isub

panel variable: isub (balanced)

. xtologit y i.tk,i(isub)

Fitting comparison model:

Iteration 0: log likelihood = -232.23571

Iteration 1: log likelihood = -230.3926

Iteration 2: log likelihood = -230.38999

Iteration 3: log likelihood = -230.38999

Refining starting values:

Grid node 0: log likelihood = -206.37423

Fitting full model:

Iteration 0: log likelihood = -206.37423

Iteration 1: log likelihood = -184.35657

Iteration 2: log likelihood = -180.62936

Iteration 3: log likelihood = -179.69436

Iteration 4: log likelihood = -179.6174

Iteration 5: log likelihood = -179.61657

Iteration 6: log likelihood = -179.61657

Random-effects ordered logistic regression Number of obs = 136

Group variable: isub Number of groups = 21

Random effects u_i ~ Gaussian Obs per group:

min = 6

avg = 6.5

max = 7

Integration method: mvaghermite Integration pts. = 12

Wald chi2(6) = 3.19

Log likelihood = -179.61657 Prob > chi2 = 0.7853

------------------------------------------------------------------------------

y | Coef. Std. Err. z P>|z| [95% Conf. Interval]

-------------+----------------------------------------------------------------

|

tk |

2 | .3986607 .6024124 0.66 0.508 -.7820459 1.579367

3 | .5276574 .7213147 0.73 0.464 -.8860934 1.941408

4 | .6579913 .6192344 1.06 0.288 -.5556858 1.871668

5 | -.0951662 .6059895 -0.16 0.875 -1.282884 1.092551

6 | .1284678 .614452 0.21 0.834 -1.075836 1.332772

7 | -.2377289 .6353866 -0.37 0.708 -1.483064 1.007606

-------------+----------------------------------------------------------------

/cut1 | -4.436589 .9839507 -4.51 0.000 -6.365097 -2.508081

/cut2 | -3.356863 .9210872 -3.64 0.000 -5.162161 -1.551565

/cut3 | -.4759493 .8391416 -0.57 0.571 -2.120637 1.168738

/cut4 | 1.405405 .8488279 1.66 0.098 -.2582675 3.069077

/cut5 | 4.380512 .9355334 4.68 0.000 2.5469 6.214124

/cut6 | 7.28738 1.230813 5.92 0.000 4.87503 9.69973

-------------+----------------------------------------------------------------

/sigma2_u | 9.825136 3.88263 4.528628 21.31624

------------------------------------------------------------------------------

LR test vs. ologit model: chibar2(01) = 101.55 Prob >= chibar2 = 0.0000

.

**Salivary cortisol and DHEA**

**Regression Analysis Models for Hormone Data**

*Cubic Splines*

Let *y* denote the log-transformed outcome (cortisol or DHEA). For each period in an analysis, the original time points (*t*) were augmented by 101 equally spaced values of *t* from 0 to 20 hours, in steps of 0.2 hours. The mean trajectory $E\left( y|t \right)$ was then modeled as a linear combination of two basis functions $U_{1}\left( t \right)$ and $U_{2}\left( t \right)$, where $U_{1}\left( t \right)=t$, and $U_{2}\left( t \right)$ is a restricted cubic spline constructed with knots at the10th, 50th, and 90th percentiles of all *t*-values (Harrell (2001)).

*Nomenclature:*

Although actual days of in-flight sample collection differed by subject, these days were grouped into three intervals, which we refer to as “periods”: early flight, mid-flight, and late flight. We also use the term “phase” to distinguish between samples gathered pre-, in, or post-flight, regardless of the actual collection day.

*Analysis Model 1. Comparing daily trajectories between the three flight periods: early (k = 1), mid (k = 2), late (k = 3) and with pre-flight (k = 0).*

Let $y_{ikj}$ denote the log-transformed hormone concentration for the *i*-th subject as measured from the *j*-th sample collection at time $t=t_{ikj}$ after awakening during the *k*-th flight period ( *j* = 1, 2, . . , *N_ik_*) . Then the mixed model for $y_{ikj}$ is given by

pre-flight (*k* = 0):

$$y_{i0j}= \mu+\beta_{1}U_{1}\left( t_{i0j} \right)+\beta_{2}U_{2}\left( t_{i0j} \right)+u_{i}+e_{i0j}$$

in-flight (*k* = 1, 2, 3):

$$y_{ikj}= \mu+\alpha_{k}+\beta_{1}U_{1}\left( t_{ikj} \right)+\beta_{2}U_{2}\left( t_{ikj} \right)+\left( \alpha\beta\right)_{k1}I_{k}U_{1}\left( t_{ikj} \right)+\left( \alpha\beta\right)_{k2}I_{k}U_{2}\left( t_{ikj} \right)+u_{i}+z_{i}+e_{ikj}$$

Parameters in this model include:

Fixed coefficients $\mu, \alpha_{k},\beta_{1}, \beta_{2},\left( \alpha\beta\right)_{k1}\left( \alpha\beta\right)_{k2}$. Here $\left( \alpha\beta\right)_{k1}$and $\left( \alpha\beta\right)_{k2}$ are differential fixed effects of the *k*-th in-flight period on $U_{1}\left( t \right)$ and $U_{2}\left( t \right)$, respectively.

Random effects

1. $u_{i}\sim N(0,\sigma_{u}^{2})$ - an overall random contribution to the intercept for the *i*-th subject.
2. $z_{i}\sim N(0,\sigma_{v}^{2})$ - a random perturbation to $u_{i}$ for all in-flight periods. This represents a random interaction between subject and flight phase (pre-flight or in-flight).
3. $e_{ikj}\sim N(0,\sigma^{2})$ - a random within-subject error term .

All random effects are modeled as mutually independent.

This model was used to decide whether there was enough information in the data to permit separate comparisons of each in-flight period with pre-flight, or whether the data from the in-flight periods should be combined and tested in aggregate against the pre-flight period.

**Results for comparison of daily log cortisol concentration trajectories**

run cort_allflight temp_cort_long zyc 0 0 3 1

Dependent variable is zyc

Model includes all in-flight periods and pre

. xtmixed zyc i.inperiod##c.U1 i.inperiod##c.U2 ||isub:phase,reml

Performing EM optimization:

Performing gradient-based optimization:

Iteration 0: log restricted-likelihood = -464.1999

Iteration 1: log restricted-likelihood = -464.1999

Computing standard errors:

Mixed-effects REML regression Number of obs = 340

Group variable: isub Number of groups = 21

Obs per group:

min = 4

avg = 16.2

max = 41

Wald chi2(11) = 71.02

Log restricted-likelihood = -464.1999 Prob > chi2 = 0.0000

-------------------------------------------------------------------------------

zyc | Coef. Std. Err. z P>|z| [95% Conf. Interval]

--------------+----------------------------------------------------------------

inperiod |

1 | .119857 .3207634 0.37 0.709 -.5088278 .7485417

2 | -.5421255 .305181 -1.78 0.076 -1.140269 .0560183

3 | -.131781 .2737346 -0.48 0.630 -.6682911 .404729

|

U1 | -.143512 .0284569 -5.04 0.000 -.1992866 -.0877375

|

inperiod#c.U1 |

1 | .0917666 .0530025 1.73 0.083 -.0121164 .1956497

2 | .1325523 .0513739 2.58 0.010 .0318614 .2332433

3 | .0875571 .0411524 2.13 0.033 .0068999 .1682143

|

U2 | .0942799 .0352814 2.67 0.008 .0251296 .1634301

|

inperiod#c.U2 |

1 | -.0944057 .064518 -1.46 0.143 -.2208587 .0320473

2 | -.1094676 .0688324 -1.59 0.112 -.2443767 .0254415

3 | -.0778542 .0500607 -1.56 0.120 -.1759714 .0202629

|

_cons | 1.397716 .1792787 7.80 0.000 1.046337 1.749096

-------------------------------------------------------------------------------

------------------------------------------------------------------------------

Random-effects Parameters | Estimate Std. Err. [95% Conf. Interval]

-----------------------------+------------------------------------------------

isub: Independent |

sd(phase) | .7974024 .1584816 .5401365 1.177204

sd(_cons) | .5045009 .1182228 .3187093 .7985997

-----------------------------+------------------------------------------------

sd(Residual) | .804882 .033028 .7426831 .87229

------------------------------------------------------------------------------

LR test vs. linear model: chi2(2) = 206.91 Prob > chi2 = 0.0000

Early Flight vs Pre-Flight

test 1.inperiod=1.inperiod#c.U1=1.inperiod#c.U2=0

chi2( 3) = 7.09

Prob > chi2 = 0.0690

Mid-Flight vs Pre-Flight

. test 2.inperiod=2.inperiod#c.U1=2.inperiod#c.U2=0

chi2( 3) = 9.59

Prob > chi2 = 0.0224

Late Flight vs Pre-Flight

. test 3.inperiod=3.inperiod#c.U1=3.inperiod#c.U2=0

chi2( 3) = 6.60

Prob > chi2 = 0.0856

Compare all 3 flight periods with each other

. test 1.inperiod=2.inperiod=3.inperiod=1.inperiod#c.U1=2.inperiod#c.U1=3.inperiod#c .U1=1.inperiod#c.U2=2.inperiod#c.U2=3.inperiod#c.U2

chi2( 8) = 12.45

Prob > chi2 = 0.1321

*Analysis Model 2. Comparing daily trajectories between the in-flight phase (φ =1) and the pre-flight phase (φ = 0).*

Model 2 was used for analysis if the results of applying Model 1 were consistent with the assumption that all three in-flight periods elicit the same response. This model has the same form as Model 1, except that the index *k* (for period) is replaced by the index *φ* (for phase) and the latter takes on only two values: 0 and 1. Here $y_{i\varphi j}$ denotes the *j*-th preflight sample if *φ* = 0, and the *j*-th in-flight sample if *φ* = 1, where for in-flight samples, $j=1, 2, ..,\sum_{k=1}^{3} N_{ik}$.

**All in-flight periods vs pre**

. run cort_more_analysis temp_cort_long zyc 0 7777777 3 1

Dependent variable is zyc

. xtmixed zyc i.phase##c.U1 i.phase##c.U2 if iss==1 & back==0 ||isub:phase ,reml nolog

Mixed-effects REML regression Number of obs = 340

Group variable: isub Number of groups = 21

Obs per group:

min = 4

avg = 16.2

max = 41

Wald chi2(5) = 64.79

Log restricted-likelihood = -455.97285 Prob > chi2 = 0.0000

------------------------------------------------------------------------------

zyc | Coef. Std. Err. z P>|z| [95% Conf. Interval]

-------------+----------------------------------------------------------------

phase |

In-flight | -.1753754 .2519939 -0.70 0.486 -.6692745 .3185236

U1 | -.1434237 .028458 -5.04 0.000 -.1992003 -.0876471

|

phase#c.U1 |

In-flight | .1022072 .0355508 2.87 0.004 .0325288 .1718855

|

U2 | .09419 .0352824 2.67 0.008 .0250378 .1633422

|

phase#c.U2 |

In-flight | -.0913781 .0440361 -2.08 0.038 -.1776873 -.0050688

|

_cons | 1.39732 .1789363 7.81 0.000 1.046611 1.748029

------------------------------------------------------------------------------

------------------------------------------------------------------------------

Random-effects Parameters | Estimate Std. Err. [95% Conf. Interval]

-----------------------------+------------------------------------------------

isub: Independent |

sd(phase) | .8045723 .1586003 .5467303 1.184015

sd(_cons) | .5020963 .1180301 .3167315 .7959446

-----------------------------+------------------------------------------------

sd(Residual) | .8049213 .0327152 .7432882 .8716651

------------------------------------------------------------------------------

LR test vs. linear model: chi2(2) = 214.15 Prob > chi2 = 0.0000

. test 1.phase=1.phase#c.U1=1.phase#c.U2=0

chi2( 3) = 11.39

Prob > chi2 = 0.0098

[Same analysis but without Subject 23]

. xtmixed zyc i.phase##c.U1 i.phase##c.U2 if iss==1 & back==0 & isub!=23 ||isub:phase ,reml nolog

Mixed-effects REML regression Number of obs = 315

Group variable: isub Number of groups = 20

Obs per group:

min = 4

avg = 15.8

max = 41

Wald chi2(5) = 75.53

Log restricted-likelihood = -412.44129 Prob > chi2 = 0.0000

------------------------------------------------------------------------------

zyc | Coef. Std. Err. z P>|z| [95% Conf. Interval]

-------------+----------------------------------------------------------------

phase |

In-flight | -.4254477 .1909258 -2.23 0.026 -.7996554 -.0512401

U1 | -.1749561 .029638 -5.90 0.000 -.2330455 -.1168666

|

phase#c.U1 |

In-flight | .1366196 .0368547 3.71 0.000 .0643856 .2088536

|

U2 | .1307289 .0380901 3.43 0.001 .0560738 .205384

|

phase#c.U2 |

In-flight | -.1375801 .0467227 -2.94 0.003 -.2291549 -.0460053

|

_cons | 1.474556 .1868746 7.89 0.000 1.108289 1.840823

------------------------------------------------------------------------------

------------------------------------------------------------------------------

Random-effects Parameters | Estimate Std. Err. [95% Conf. Interval]

-----------------------------+------------------------------------------------

isub: Independent |

sd(phase) | .2994589 .142239 .1180396 .7597079

sd(_cons) | .5365314 .1184366 .3480941 .8269772

-----------------------------+------------------------------------------------

sd(Residual) | .8006416 .033746 .7371589 .8695913

------------------------------------------------------------------------------

LR test vs. linear model: chi2(2) = 83.72 Prob > chi2 = 0.0000

Note: LR test is conservative and provided only for reference.

. test 1.phase=1.phase#c.U1=1.phase#c.U2=0

( 1) [zyc]1.phase - [zyc]1.phase#c.U1 = 0

( 2) [zyc]1.phase - [zyc]1.phase#c.U2 = 0

( 3) [zyc]1.phase = 0

chi2( 3) = 15.36

Prob > chi2 = 0.0015

.

*Analysis Model 3. Comparing daily trajectories between either recovery period; early (k =4), or late (k = 5), and the pre-flight period (k = 0).*

This model was fit separately to compare trajectories for early recovery vs. pre-flight, and also for late recovery vs. pre-flight. Model 3 has the same form as Model 1, except that the values of *k* are now 0 (pre-flight), and either 4 or 5 (post-flight).

**Early Recovery vs Pre-flight**

. xtmixed zyc i.period##c.U1 i.period##c.U2 if iss==1 & back==0 ||isub:phase ,reml nolog

Mixed-effects REML regression Number of obs = 190

Group variable: isub Number of groups = 21

Obs per group:

min = 2

avg = 9.0

max = 17

Wald chi2(5) = 61.57

Log restricted-likelihood = -270.56062 Prob > chi2 = 0.0000

------------------------------------------------------------------------------

zyc | Coef. Std. Err. z P>|z| [95% Conf. Interval]

-------------+----------------------------------------------------------------

period |

Post Early | -.0547662 .2795901 -0.20 0.845 -.6027527 .4932204

U1 | -.1445594 .0315748 -4.58 0.000 -.206445 -.0826739

|

period#c.U1 |

Post Early | .0654288 .0558411 1.17 0.241 -.0440177 .1748753

|

U2 | .0977262 .0393991 2.48 0.013 .0205054 .174947

|

period#c.U2 |

Post Early | -.1283149 .081322 -1.58 0.115 -.2877032 .0310733

|

_cons | 1.421559 .1764032 8.06 0.000 1.075815 1.767303

------------------------------------------------------------------------------

------------------------------------------------------------------------------

Random-effects Parameters | Estimate Std. Err. [95% Conf. Interval]

-----------------------------+------------------------------------------------

isub: Independent |

sd(phase) | .2679939 .0955772 .1332149 .5391344

sd(_cons) | .4114733 .1132772 .2398882 .7057884

-----------------------------+------------------------------------------------

sd(Residual) | .8770741 .050343 .7837515 .9815088

------------------------------------------------------------------------------

LR test vs. linear model: chi2(2) = 18.09 Prob > chi2 = 0.0001

. test 4.period=4.period#c.U1=4.period#c.U2=0

chi2( 3) = 2.83

Prob > chi2 = 0.4187

**Late Recovery vs Pre-flight**

. xtmixed zyc i.period##c.U1 i.period##c.U2 if iss==1 & back==0 ||isub:phase ,reml nolog

[output not shown]

. test 5.period=5.period#c.U1=5.period#c.U2=0

chi2( 3) = 7.90

Prob > chi2 = 0.0481

All models were fit using the method of restricted maximum likelihood, which has been shown to provide more accurate inference than maximum-likelihood when sample sizes are small (Diggle, Liang and Zeger (1995)). Inference on the effect of flight or recovery relative to pre-flight on daily trajectories was made using Wald tests.

**Results for comparison of daily log DHEA concentration trajectories**

Analysis models were the same as for log cortisol concentration.

. run cort_allflight temp_cort_long zyd 0 7777777 3 1

Dependent variable is zyd

. xtmixed zyd i.period##c.U1 i.period##c.U2 if phase<=1 & iss==1 & back==0 ||isub:phase ,reml nolog

[Analysis output not shown – similar to that for log cortisol]

Early Flight vs Pre-Flight

. test 1.period=1.period#c.U1=1.period#c.U2=0

chi2( 3) = 2.14

Prob > chi2 = 0.5432

Mid-Flight vs Pre-Flight

. test 2.period=2.period#c.U1=2.period#c.U2=0

chi2( 3) = 0.46

Prob > chi2 = 0.9286

Late Flight vs Pre-Flight

. test 3.period=3.period#c.U1=3.period#c.U2=0

chi2( 3) = 0.03

Prob > chi2 = 0.9984

**All in-flight periods vs pre**

. xtmixed zyd i.phase##c.U1 i.phase##c.U2 if iss==1 & back==0 ||isub:phase ,reml nolog

[output not shown]

. test 1.phase=1.phase#c.U1=1.phase#c.U2=0

chi2( 3) = 0.27

Prob > chi2 = 0.9654

Early Recovery vs Pre-Flight

. xtmixed zyd i.period##c.U1 i.period##c.U2 if iss==1 & back==0 ||isub:phase ,reml nolog

[output not shown]

test 4.period=4.period#c.U1=4.period#c.U2=0

chi2( 3) = 3.51

Prob > chi2 = 0.3192

Late Recovery vs Pre-Flight

. xtmixed zyd i.period##c.U1 i.period##c.U2 if iss==1 & back==0 ||isub:phase ,reml nolog

[output notshown]

. test 5.period=5.period#c.U1=5.period#c.U2=0

chi2( 3) = 3.95

Prob > chi2 = 0.2664

.

**Example of Model Fit and Residual Analysis**

As an example, after fitting Model 2, with log cortisol concentration as the dependent variable Figures 1 and 2 show the estimated mean daily trajectory of log cortisol concentration for pre-flight and in-flight samples, respectively. Superimposed are the original data (gray) and adjusted data, which is the original data with the best-linear-unbiased (B.L.U.P.) predicted values of the subject-level random effects removed (solid dots). As described above, we found a significant effect of flight on these trajectories:

. test 1.phase=1.phase#c.U1=1.phase#c.U2=0

chi2( 3) = 11.39

Prob > chi2 = 0.0098

Figure1. Estimated Mean Daily Trajectory for Pre-flight Samples

Figure 2. Estimated Mean Daily Trajectory for In-flight Samples.

Figures 3-5 show q-q plots of the three types of residuals corresponding to the three types of best linear unbiased predictors of $e_{ikj}$, $u_{i}$, and $z_{i}$, respectively. It can be seen that normality assumptions for $e_{ikj}$ and $u_{i}$ are quite good, however the assumption of normality of $z_{i}$ is not well satisfied because of one outlier subject (“X”). Inclusion of this subject biases the estimate towards zero and inflates the standard error of the in-flight main effect coefficient and thus reduces the power to detect a phase effect. Without this subject in the analysis, the test for an overall phase effect produces a chi-squared value of 15.4 (p = 0.0015), as compared with 11.4 (p = 0.0098) with the subject included. The q-q plot for the $z_{i}$ in the revised analysis is shown in Fig. 6.

Figure 3. q-q plot for Lowest-level Residuals ($e_{ikj}$) All Subjects

Figure 4. q-q plot for Subject-level Random Intercepts ($u_{i}$). All Subjects.

Figure 5. q-q plot for Subject-level Random Interactions ($z_{i}$). All Subjects.

Figure 6. q-q plot for Subject-level Random Interactions ($z_{i}$). Subject 23 removed.

*References*

Diggle, P., Liang, K. Y. and Zeger, S. L. 1995. *Analysis of Longitudinal Data.* Oxford Science Publications, Clarendon Press: Oxford. pp.64-68.

Harrell, F. E., Jr. 2001. Regression Modeling Strategies: With Applications to Linear Models, Logistic Regression,

and Survival Analysis. New York: Springer.
